# Abattoirs as Non-Hospital Source of Extended Spectrum Beta Lactamase Producers: Confirmed by the Double Disc Synergy Test and Characterized by Matrix-Assisted Laser Desorption/Ionization Time of Flight Mass Spectrometry

**DOI:** 10.1371/journal.pone.0094461

**Published:** 2014-04-11

**Authors:** Moses Nkechukwu Ikegbunam, Linda Onyeka Anagu, Ifeanyi R. Iroha, Chika Ebiye Ejikeugwu, Charles Okey Esimone

**Affiliations:** 1 Departments of Pharmaceutical microbiology and Biotechnology, Faculty of Pharmaceutical Sciences, Nnamdi Azikiwe University, Awka, Anambra State, Nigeria; 2 Department of Applied Microbiology, Faculty of Biological Sciences, Ebonyi State University, Abakaliki, Ebonyi State, Nigeria; Aix Marseille Université, France

## Abstract

In this study, the presence of extended spectrum beta lactamase (ESBL) producing organisms in abattoirs, a non-hospital community was investigated. The presence of ESBL-producing phenotypes was confirmed by the Double Disc Synergy Test (DDST). Out of the 99 isolates screened for ESBL, 28 (28.3%) were confirmed positive. The positive isolates were characterised by using Matrix-Assisted Laser Desorption/Ionization Time of flight Mass Spectrometry. 50% of the isolates were *Pseudomonas spp*., the rest were different species of *Acinetobacter, Stenotrophomonas and Achromobacter*. *Pseudomonas monteilli and Pseudomonas putida* were the most occurring in the intestine. The entire positive ESBL producers were subjected to plasmid curing to ascertain the location of the resistant marker. The result of the plasmid curing indicated that the resistant genes were chromosomally borne. The findings have therefore established the presence of ESBL producing organisms in the gut of animals from abattoirs and the table were the meat are sold, and its rate of occurrence is comparable to hospital ICUs. Abattoir communities could probably be a source of human infection with ESBL expressing pathogens and possible transfer to non-ESBL producers.

## Introduction

Extended spectrum Beta Lactamases (ESBL) producers are a group of Gram-negative bacteria that produce a kind of β-lactamase enzyme, called ESBL that hydrolyze a broader spectrum of β-lactam antibiotics (including both penicillins and cephalosporins) than that hydrolyzed by the simple β-lactamases. Simple β-lactamases hydrolyze mainly the Narrow spectrum β-lactam antibiotics. ESBL have ability to inactivate β-lactam antibiotics containing an oxyimino-group such as oxyimino-cephalosporins (e.g., ceftazidime, ceftriaxone, cefotaxime) as well as oxyimino- monobactam (aztreonam). [1], [2] They are not active against cephamycins and carbapenems. Generally, they are inhibited by β-lactamase-inhibitors such as clavulanic acid and tazobactam. The genes that encode for these enzymes may be plasmid-borne or chromosomally located.

ESBL has only been detected in Gram-negative rods, mainly species of the *Enterobacteriaceae* family, and they can be acquired from a number of sources. Exposure to ESBL producing microorganisms can occur through any means but the hospital has always been thought to be the greatest risk, especially in intensive care units (ICUs). [3], [4] Recent studies have demonstrated the danger of ESBL producers in livestock. [1]

The occurrence of ESBL producing microorganisms is on the rise globally, with prevalence varying from country to country and within a country from institution to institution. A survey on 81,213 bloodstream infectious pathogens during 1997–2002 showed that the *Klebsiella spp*. with an ESBL phenotype was isolated at a rate of 42.7% in Latin America, 21.7% in Europe and 5.8% in North America. [5] In Nigeria, there have been reports of ESBL producing organisms with documentations particularly in the western [6] and in the eastern part of the country from both secondary and tertiary institutions. [7], [8] The presence of ESBL can be detected by phenotypic methods and molecular methods. Phenotypic method is based upon the resistance that ESBLs confer to oxyimino- β- lactams (e.g ceftriaxone, cefotaxime, ceftazidime and aztreonam) and the ability of a β-lactamase inhibitor, usually clavulanic acid, to block this resistance. One of such phenotypic methods is the double disk diffusion test.

Non-hospital sources of ESBL microorganisms are important because of the low level of hygiene and little or no aseptic techniques that is achievable in such institutions. Here, we investigate abattoirs as a potential non-hospital source of ESBL microbes. This was achieved and underscores the need for proper hygiene among livestock keepers, meat sellers and buyers, and in handling and cooking meat at home.

## Materials and Method

### Test Microorganism

99 bacterial isolates gotten from swab samples of the intestine of animals killed on the spot and of the surfaces (fomites) of tables where the meat are sold, in an abattoir in Agu Awka, Anambra state, Nigeria, were used in this study. The isolation was done using Nutrient agar and MacConkey agar. Ethical approval for obtaining such specimen was not required since the animals were killed by the meat sellers for the purpose of their trade. But, we obtained their oral consent after having explained to them what we were to use the swab samples for.

### Screening for ESBL Isolates

This was determined by using the double disc synergy test (DDST). Here, a 20 ml volume of Mueller Hinton agar was prepared and dispensed aseptically into each of 60 Petri dishes. A 0.1 ml suspension of each of the isolates equivalent to 0.5 ml MacFarland standard was aseptically seeded into the Petri dishes together with Muller Hinton agar. This was allowed to stand for 1 hour to solidify. A combination disc (Amoxicillin 20 µg and Clavulanic acid 10 µg) was placed at the centre of the Petri dish and antibiotics; Ciprofloxacin 30 µg and Cefuroxime 30 µg or Ceftaxidine or Cefotaxime were placed 15 mm apart centre to centre on both sides of the plates.

The set up was done in triplicate and it was left for 30 minutes to pre-diffuse into the medium. It was incubated at 37°C for 24 hours after which the various inhibition zone diameters were measured.

### Characterization of ESBL producing Abattoir Isolates

The Matrix-Assisted Laser Desorption/Ionization Time of flight Mass Spectrometry (MALDI-TOF MS) was used to identify the isolates to the specie level. Briefly, a few colonies of fresh overnight cultures grown on blood agar plates at 37°C under aerobic conditions were suspended in 600 µl of 70% ethanol in eppendorf tube and vortex for 1 min to mix. This was centrifuged for 3 minutes at 13,000 rpm and the supernatant was poured away leaving the pellet. 20 µl of formate was added to the pellet mixed properly and vortex, 20 µl of aceto-nitrile was added into the mixture and vortex to mix. The mixture was centrifuged for 1 min at 13,000 rpm; after which 1 ml of the sample was gently dropped on the target plate and was allowed to air dry. After drying, 1 ml of the matrix solution was added and again air dried at room temperature.

MALDI-TOF measurements were performed with a bruker 2.0 Ultraflex II MALDI-TOF MS (Bruker Daltonik GmbH, Bremen, Germany) instrument equipped with 200 H_Z_ smartbeam laser technology was used for visual inspection of mass spectra. The whole process from MALDI-TOF MS measurement for identification was performed automatically. The spectra were recorded in the linear positive mode at a laser frequency of 20 Hz within a mass range of 2,000 to 20,000 Da with 50 shots per second from different positions of the target spot.

To identify unknown bacterial isolates, raw spectra were imported into MALDI Biotype software and analysed by the standard pattern matching algorithm against the library spectra using the standard settings. In pattern matching's, fingerprints of unknown samples were compared to all entries of the data base (Bruker Biotype database). Results of the pattern matching process were expressed as log (score) values ranging from 0 to 3. Values of >1.7 generally indicates relationships on the genus level and log (scores) of >2.0 relationships on the species level. The highest log (score) of a match against the database was used on the species identification. Each of the characterized isolates was stored. They were sub-cultured at 37°C for 18–24 hr and standardized to 1×10^5^ cfu/ml prior to any microbiological assay.

### Plasmid Curing

The sensitivity of each isolate was determined using different antibiotics. ESBL producing isolates were grown in Mueller Hinton broth and diluted in Double Strength Mueller Hinton broth. Five test tubes of diluted organism for each isolate. A determined concentration and volume of acridine orange solution in sterile distilled water was added to the different test tube such that the resulting concentration of the acridine orange was 0.1 mg/ml, 0.2 mg/ml, 0.3 mg/ml, 0.4 mg/ml, 0.5 mg/ml; representing the five different test tubes; and the Muller Hinton broth was single strength. All the test tubes were incubated at 37°C for 24 hr. The sensitivity of the isolates in the test tubes was determined after incubation.

## Results and Discussion

### Screening for ESBL Isolates

There was a high ESBL production with 28 out of 99 of the isolates being positive for ESBL production as shown in Table S1. That is, 28.3% of the isolates are ESBL producers. 11 of these ESBL producers were gotten from fomites, as explained above. The isolates showed the synergistic symbol that is characteristic of ESBL production as seen in appendix I. This is also confirmatory of the double disc test for the detection of ESBL producing organisms as proposed by Brun Buisson et al, (1989).[9] This high occurrence of ESBL producers is comparable to that in the hospital ICUs. [10]

### Characterization of ESBL Isolates

The characterization of the test isolates using the Maldi-Tof technique identified different species of *Pseudomonas, Acinetobacter, Stenotrophomonas and Achromobacter* (Table S1). 50% of the isolates were *Pseudomonas spp*. Other organisms isolated were different species of *Acinetobacter, Stenotrophomonas and Achromobacter*. Most of these organisms isolated have been implicated in several human diseases and there is a possibility of contacting these organisms from improperly processed cow meats.

Several of them are environmental bacteria but have been implicated in human infection e.g. first case of infection of the cerebrospinal fluid of a human by *Pseudomonas fulva*; an environmental bacterium. [11] Nosocomial infections due to Multidrug-Resistant isolates of *Pseudomonas putida* producing VIM-1 Metallo-β-lactamase has been reported. [12] There is a first report of bacteraemia caused by *Pseudomonas fulva* has been published. [13] There is another case where *Pseudomonas mendocina* was isolated from a patient with infective endocarditis. [14]

*Achromobacter species* have been implicated in bacteremia in 46 patients with cancer from 1989 to 2003. [15] *Acinetobacter baumanii*, found ubiquitously in the environment, is an aerobic gram negative rod which is a non-fermenter of glucose. Multidrug resistant *Acinetobacter baumanii* is an important cause of hospital acquired infection and has been shown in some studies to increase mortality and length of stay. [16]

*Stenotrophomonas* infections have been associated with high morbidity and mortality in severely immunocompromised and debilitated individuals. *S. maltophilia* colonization rates in individuals with cystic fibrosis have been increasing. [17] Though *Stenotrophomonas* species are environmental bacteria, but when contacted by human could be very difficult to eradicate. They are naturally resistant to many broad-spectrum antibiotics (including carbapenems). [18]

### Plasmid Curing

The drug resistant marker of an organism could be chromosomal or extra chromosomal. [19] Table? 1 shows the sensitivity of the isolates before and after plasmid curing. The sensitivity is measured as the inhibition Zone Diameter (IZD) in millimetres.

**Table 1:** Pre and Post Plasmid Curing Sensitivity Test (IZD* in mm).

	GEN		ERY		TET		CAZ		AMX	
ISOLATES	Pre	Post	Pre	Post	Pre	Post	Pre	Post	Pre	Post
*P. monteilli*	12	11	12	10	10	10	0	0	0	0
*P. putida*	22	21	12	16	10	0	0	0	0	0
*P.beteli*	21	18	12	0	13	14	0	0	0	0
*P. mendocina*	20	19	11	9	12	13	0	0	0	0
*P. putida*	19	18	11	0	12	0	0	0	0	0
*P. monteilli*	20	19	11	9	12	13	0	0	0	0
*P. monteilli*	12	11	12	10	10	10	0	0	0	0
*P*. *Putida*	20	19	11	0	12	0	0	0	0	0
*P. fulva*	20	20	11	10	12	14	0	0	0	0
*P. mendocina*	20	19	11	9	12	13	0	0	0	0

*Each value represents the mean value of two determinants IZD (mm). GEN =  Gentamicin, ERY =  Erythromycin, TET =  Tetracycline, CAZ =  Ceftazidime, AMX =  Amoxicillin.

A close observation would reveal that the isolates that tested positive for ESBL were resistant to CAZ and AMX after treatment with acridine orange at different concentration. This result concluded that the resistance marker is not plasmid borne but chromosomal. It was also observed that the positive isolates treated with 0.5 mg/ml of acridine orange were inhibited. This revealed the effect of acridine orange against bacterial at higher concentration. Summarily, none of the resistant markers were lost. All the organisms retained the resistant markers. This suggests that the resistant markers are of chromosomal origin. [6]

In a similar experiment carried out by Yah et al, (2007) [20] at the Ahmadu Bello University teaching hospital (ABUTH) Zaria, the plasmid curing experiment showed that some bacteria were sensitive while others were still resistant. The susceptibility of the organisms was indicative of plasmid mediated resistance while growth in the Mueller Hinton again was indicative of chromosome – mediated resistance.

In his study, Enabulele et al (1993) [21], reported that the resistance of Gram negative bacteria isolated from infected wounds from the University of Benin Teaching Hospital was plasmid mediated since the organism became sensitive after curing.

## Conclusion

This study revealed the presence of ESBL producing organisms that can be opportunistic or potential pathogens in Abattoirs. These organisms also occurred at a high rate comparable to hospital ICUs. *Pseudomonas spp*. had the greatest occurrence; with *Pseudomonas monteilli and Pseudomonas putida* being the most abundant in the intestine. There is thus a high rate of antibiotic misuse among animal keepers. Although, the ESBL resistant markers in these organisms are chromosomally borne, the occurrence of high frequency recombinant strains can still lead to easy transfer of such resistant traits to non-ESBL producers within a give niche. Proper hygiene in abattoir among buyers and sellers is of primary importance.

## Supporting Information

Table S1 Phenotypic Confirmation of ESBL Production Using DDST.
(DOCX)
