# Supplementary material for: Abattoirs as Non-Hospital Source of Extended Spectrum Beta Lactamase Producers: Confirmed by the Double Disc Synergy Test and Characterized by Matrix-Assisted Laser Desorption/Ionization Time of Flight Mass Spectrometry
Source: PLoS One. 2014 Apr 11;9(4):e94461. doi: 10.1371/journal.pone.0094461 (PMC3984184; doi:10.1371/journal.pone.0094461)
Supplement: Table S1 — Phenotypic Confirmation of ESBL Production Using DDST. (DOCX) [file pone.0094461.s001.docx]

SUPPORTING INFORMATION

**Table S1: Phenotypic Confirmation of ESBL Production Using DDST**

**Inhibition Zone Diameter of Antibiotics (mm)**

| **ISOLATES** | **CAZ** | **CAZ+AMC** | **CTX** | **CTX + AMC** |
| --- | --- | --- | --- | --- |
| \| *Pseudomonas monteilli* (F1),  *Acinotobacter baumannii* (F1)*,*  *Acinetobacter baumannii* (I1)*,*  *Stenotrophomonas maltophilia* (I1)*,*  *Stenotrophomonas nitritireducens* (F1)*,*  *Achromobacter ruhlandii* (I1),  *Acinetobacter baumannii* (I2)*,*  *Pseudomonas putida* (F1)*,*  *Stenotrophomonas maltophilia* (I2)*,*  *Stenotrophomonas maltophilia* (F1)*,*  *Pseudomonas beteli* (F1)*,*  *Stenotrophomonas maltophilia* (F2) *,*  *Pseudomonas mendocina* (I1)*,*  *Pseudomonas putida* (I1)*,*  *Acinetobacter baumannii* (F2)*,*  *Pseudomonas monteilli* (I1)*,*  *Pseudomonas monteilli* (I2)*,*  *Pseudomonas putida* (I2)  *Pseudomonas monteilli (*I3*).*  *Pseudomonas fulva* (F1)  *Achromobacter ruhlandii* (I2)  *Pseudomonas putida* (I3)  *Pseudomonas monteilli* (F2)  *Achromobacter ruhlandii* (F1)  *Stenotrophomonas nitritireducens* (F2)  *Pseudomonas fulva* (I2)  *Pseudomonas mendocina* (I2)*,*  *Acinotobacter baumannii (I2)* \| \| --- \| | 0  0  21  14  0  0  0  21  19  7  17  17  11  15  16  0  15  19  0  21  25  24  0  0  0  21  0  16 | 29  28  27  29  23  27  20  28  27  29  28  27  20  28  29  31  33  29  30  34  25  28  31  35  25  29  22  32 | 0  0  20  0  0  0  0  0  20  18  0  16  13  0  11  0  0  11  13  0  12  11  0  18  0  14  0  17 | 30  23  21  20  25  26  19  22  26  29  28  24  27  21  20  23  22  25  29  24  31  30  24  28  21  29  22  32 |

**KEYS**

**CAZ- Ceftazidime; CTX- Cefotaxime; AMC- Amoxicillin+ Clavulanic acid; F – Formites; I – Intestine, 1,2,3 denoting the isolate number.**
